# Individual differences in the experience of body ownership are related to cortical thickness

**DOI:** 10.1038/s41598-021-04720-8

**Published:** 2022-01-17

**Authors:** Timea Matuz-Budai, Beatrix Lábadi, Eszter Kohn, András Matuz, András Norbert Zsidó, Orsolya Inhóf, János Kállai, Tibor Szolcsányi, Gábor Perlaki, Gergely Orsi, Szilvia Anett Nagy, József Janszky, Gergely Darnai

**Affiliations:** 1 Institute of Psychology, University of Pécs, 6 Ifjúság str., Pécs, 7624 Hungary; 2 Institute of Philosophy and Art Theory, University of Pécs, Pécs, Hungary; 3 Department of Behavioural Sciences, Medical School, University of Pécs, Pécs, Hungary; 4 Department of Neurology, Medical School, University of Pécs, Pécs, Hungary; 5 MTA-PTE, Clinical Neuroscience MR Research Group, Pécs, Hungary; 6 Pécs Diagnostic Centre, Pécs, Hungary; 7 Department of Neurosurgery, Medical School, University of Pécs, Pécs, Hungary; 8 Neurobiology of Stress Research Group, Szentágothai Research Centre, University of Pécs, Pécs, Hungary; 9 Department of Laboratory Medicine, Medical School, University of Pécs, Pécs, Hungary

**Keywords:** Human behaviour, Somatosensory system

## Abstract

The widely used rubber hand illusion (RHI) paradigm provides insight into how the brain manages conflicting multisensory information regarding bodily self-consciousness. Previous functional neuroimaging studies have revealed that the feeling of body ownership is linked to activity in the premotor cortex, the intraparietal areas, the occipitotemporal cortex, and the insula. The current study investigated whether the individual differences in the sensation of body ownership over a rubber hand, as measured by subjective report and the proprioceptive drift, are associated with structural brain differences in terms of cortical thickness in 67 healthy young adults. We found that individual differences measured by the subjective report of body ownership are associated with the cortical thickness in the somatosensory regions, the temporo-parietal junction, the intraparietal areas, and the occipitotemporal cortex, while the proprioceptive drift is linked to the premotor area and the anterior cingulate cortex. These results are in line with functional neuroimaging studies indicating that these areas are indeed involved in processes such as cognitive-affective perspective taking, visual processing of the body, and the experience of body ownership and bodily awareness. Consequently, these individual differences in the sensation of body ownership are pronounced in both functional and structural differences.

## Introduction

The growing body of neurocognitive findings surrounding self-location, agency, and body ownership has emphasized the role of multisensory processes that may contribute to bodily self-consciousness. Previous research on bodily self-consciousness have studied how bodily illusions can modulate the experience of one’s ownership over their own body, referring to the feeling that the body is an integral part of the self^1–3^.

The most commonly used experimental paradigm to manipulate body ownership is the rubber hand illusion (RHI). During the illusion, the synchronous stroking of a rubber hand and the participant’s own hand, the latter of which is separated from their visual field, leads the participants to perceive that the rubber hand is part of their own body^1,3,4^. However, the RHI does not occur when the rubber hand and the participant’s own hand are stroked asynchronously^1,3^, or when participants view the rubber hand in an anatomically implausible position (e.g., watching a right rubber hand while their left hand is being stimulated), or when viewing a neutral object, even though the stimulation is synchronous^5^. The RHI is a useful tool for measuring both the objective and the subjective aspects of body ownership. The objective (or implicit) measure of the RHI is the proprioceptive drift*,* which refers to the degree of displacement of the perceived position of the participant’s real hand towards the rubber hand. Conversely, the subjective (or explicit) aspect of the RHI refers to the phenomenological experience of the illusion and is usually measured by self-report questionnaires^6^.

With respect to the relationship between proprioceptive drift and subjective changes in body ownership during the RHI, however, the literature shows contradictory results. Initial studies of the RHI found that the proprioceptive drift was positively associated with the subjective feeling of owning the rubber hand, suggesting common brain mechanisms for illusory hand ownership and proprioceptive drift^4,5,7^. However, other studies revealed that the proprioceptive drift and the sense of body ownership are rarely correlated (e.g.^8^), suggesting there are distinct multisensory mechanisms underlying the phenomena. These findings have been further supported by functional magnetic resonance imaging (fMRI) studies demonstrating that there are at least two components of bodily self-consciousness: body ownership and self-location (i.e., the physical location of the body in a space)^9^. Furthermore, other functional brain imaging studies investigating various bodily illusions such as RHI^1,10,11^, full body illusion^12^, and body swap illusion^13^ also found convergent evidence for the notion that bodily self-consciousness is represented in a distributed neural network in the brain, including the premotor cortex (PMC), the parietal area, the somatosensory cortex, the occipitotemporal cortex, and the insula.

In line with this, a wide range of neurocognitive processes have been shown to be involved in the RHI. First, the body-related sensory information is processed in a bottom-up way in the sensory regions. With regard to the neurocognitive mechanism underlying bodily illusions, visual information relating to the body and body parts initially activate the occipital cortex*,* mainly the extrastriate body area (EBA^14^), which is selectively active when body parts are visually perceived^15^. The tactile sensation felt on the skin/body activates the primary somatosensory cortex in the postcentral gyrus^16,17^, while the proprioceptive information stimulates the precentral gyrus, the postcentral gyrus, and the anterior cingulate gyrus^18^. Although these unimodal visual, tactile, and proprioceptive body representations are essential components of the bodily experience, multisensory processes are also necessary. Multisensory areas in premotor, parietal, and insular cortices integrate sensory cues originating from various modalities and it has even been suggested that they provide the whole-body experience. One of the first fMRI studies targeting body ownership and the RHI^1^ postulated that the self-attribution of body parts arises as a result of multisensory integration processes in the PMC*.* More specifically, the increased activity in the bilateral ventral PMC (vPMC) has been associated with the feeling of ownership over the observed fake hand. Another fMRI study using the body swap illusion (viewing the body of a mannequin from its head’s point of view receiving tactile stimulation while the participant’s body is out of sight and is stimulated identically to the mannequin) also demonstrated that neural activity in the vPMC correlated with the strength of the experienced ownership of a virtual body, and also of a virtual hand^13^. Thus, these results suggest that the increased activation of vPMC reflects the subjective feeling of body ownership over a fake body part (e.g., a prosthetic hand) resulting from the integration of multisensory cues from various sources.

Previous studies showed that the temporo-parietal junction region (TPJ) involving the supramarginal gyrus (SMG) and the angular gyrus also contributes to the top-down process of the internal representation of the body, as it can affect the structural model thereof in order to facilitate the experience of ownership over one’s own body. Moreover, TPJ activity also modulates the converging visual, proprioceptive, vestibular, and tactile signals in order to construct a world-centered reference frame which provides stable self-location and visuo-spatial perspective^19,20^. The posterior parietal cortex (PPC) also plays a critical role in multisensory integration, transforming the sensory inputs from the body (i.e., information about the hands, arms, head, etc.) into a body map, which obtains a spatially represented body-centred reference frame. This mechanism contributes to the internal representation of self-location^2,10^. Perceiving a rubber hand as one’s own during an experimental illusion seems to result from a remapping process in PPC indicating changes in the sense of hand position, whilst the hand remapping in the PMC is related to the subjective feeling of body ownership^10^. Other studies investigating the neural basis of the bodily self have discovered that the activation in the right insula were positively correlated with the strength of the RHI^3^. The insula is known as a structure that integrates bodily signals with environmental information in order to increase homeostatic efficiency^21^; this plays a vital role in interoceptive and exteroceptive awareness (for a review, see^22^). Based on the converging findings, it is logical to hypothesize there exists a multicomponent neural network that is responsible for the modifications in bodily self-consciousness evoked by bodily illusion paradigms.

However, this neural model cannot explain the findings of previous studies demonstrating that 30% of the population do not experience („non-perceivers”) body ownership over the fake hand^7,23^. This suggests that individual differences exist in bodily self-consciousness. For example^7^, proposed that people who are better at relying on proprioceptive information (e.g., professional dancers) might be more resistant to the illusion than others. In line with this^24^, found evidence that subjects with lower interoceptive awareness were more susceptible to the experience of the ownership of corporeal objects (fake hands) in the RHI experiment. Similarly, sensory suggestibility is likely to contribute to the high response variability in RHI findings. A study by^25^ compared individuals with high and low sensory suggestibility and ascertained that highly suggestible individuals had a stronger experience of body ownership over the rubber hand, as measured by a subjective questionnaire, while the results of the implicit measures (proprioceptive drift) implied no differences between the two groups. For a deeper understanding of the individual differences found in this bodily illusion, a recent study attempted to investigate the relationship between brain anatomy and subjective reports of functional self-experience (measuring body ownership, narrative self and agency) in healthy individuals^26^. A significant association was observed between the insular cortex volume and ownership-related self-malfunctions (such as schizotypal, depersonalizing, and dissociative tendencies) in daily life. More specifically, the ownership subscale scores correlated with grey matter volumes in the postcentral gyrus, insular cortex, and angular gyrus, thus indicating that individual differences in cortical volume are related to ownership experiences, leading to the assumption that the morphology of certain brain structures could explain variabilities in body ownership experiences reported following the induction of a bodily illusion. However, this study assessed the body ownership and other self-related functions in an imagined daily situation. Furthermore, several studies showed altered cortical thickness, volume and cortical surface in body ownership-related brain areas in disorders where the representation of the body is impaired. In case of body integrity identity disorder (also known as xenomelia) a study found reduced cortical thickness in the superior parietal lobule and reduced cortical surface area in the anterior insular cortex, the somatosensory areas SI and SII, and the inferior parietal lobule^27^. In addition, several lines of evidence suggest that patients with anorexia nervosa have alterations in cortical thickness with respect to the stages of weight restoration. Comparing with healthy controls^28^, found globally lower cortical thickness in women with anorexia nervosa. In a longitudinal study it was shown that three months of weight restoration resulted in the normalization of cortical thickness in patients with anorexia nervosa^29^. Studies also reported increased gray matter volume of the medial orbitofrontal cortex and the insula^30^, and greater cortical thickness of the medial orbital sulcus and the insula^31^ in patients with anorexia nervosa.

To the best of our knowledge, no research has been conducted to shed light on the structural neuroanatomical differences that might explain the robust inter-individual differences in the RHI. Here, we aim to study whether the individual differences of body ownership experience in the RHI could be explained by structural brain differences in terms of cortical thickness. Cortical thickness was chosen primarily for methodological reasons. Firstly, it was validated in histological studies and it was found as a method with high accuracy (e.g.^32^). Secondly, Freesurfer’s thickness estimation method was tested on several clinical samples (e.g. schizophrenia^33,34^, Huntington’s disease^35,36^, epilepsy^37,38^), elderly people and young adults. These studies confirmed that this measurement tool provides a sensitive marker for clinical symptoms and psychological variables as well. We hypothesized that the strength of the proprioceptive drift and the subjective report of body ownership are associated with the cortical thickness of specific right hemispheric brain areas involved in the multisensory integration process: superior temporal gyrus (STG^39,40^), middle temporal gyrus (MTG^41^), inferior temporal gyrus^42^, precuneus^39,40^, SMG^19,20^, precentral and postcentral gyri^18^, superior parietal gyrus^43,44^, inferior parietal gyrus^45^, lateral and medial orbitofrontal cortices (OFC^46^), rostral anterior cingulate cortex^18^, pars opercularis^45,47,48^, lateral occipital cortex (LOC^14^), and insula^3^.

## Methods

### Participants

67 university students (36 were male) participated in this experiment, with a mean age of 22.25 years (SD: 2.29, range 19–29 years). All participants were right-handed according to the Edinburgh Handedness Inventory (i.e., the Handedness Laterality Quotient for each participant was higher or equal to 70%^49^). Participants did not have a history of neurological diseases or experience with the RHI. Informed consent was obtained prior to the experimental sessions. All participants received a small fee for taking part in the study. The study was conducted according to the principles of the Declaration of Helsinki and had been approved by the Regional Research Ethics Committee of the Medical Center, Pécs.

### Experimental design and procedures

#### Rubber hand illusion paradigm

In this study, we used the original RHI paradigm introduced by^4^, supplemented with a proprioceptive drift baseline measurement before the stimulation. During the experiment, participants were sitting on a comfortable chair and their arms rested on a table with their palms facing down. A realistic artificial hand (left or right rubber hand, depending on which hand was stimulated) was placed on the table 20 cm from the participant’s real hand. In case of the non-stroked hand, we asked the participants to rest their hand on the table comfortably in a symmetrical position to the stroked hand. Only the rubber hand was visible to the participants, whereas both of their own hands were hidden behind a 40 cm × 65 cm wooden occluder. The RHI was performed by a trained experimenter who stroked both the real hand and the rubber hand with paintbrushes for two minutes while the subject was instructed to focus on the rubber hand. The experimenter stroked the upper and the lower part separately of all fingers (except the thumb) on the rubber and the unseen hand with two brushes at the same time. The illusion was elicited on both hands in a counterbalanced order across participants. The experimental block for both hands consisted of a baseline proprioceptive drift measurement (without stroking period) and two experimental conditions (synchronous and asynchronous stimulation). The experimental conditions were also counterbalanced across the blocks. One stroking period per condition was performed.

### Procedure

Before the stimulations, participants underwent a baseline proprioceptive drift measurement in which the experimenter measured the pre-stroking difference between the actual and the perceived location of the hand. The experimenter asked the participant to close their eyes and point to the location of their real index finger on a ruler. A positive value for proprioceptive drift indicated that the participants perceived the location of their own index finger as drifting toward the rubber hand (toward the body midline). After the baseline measurement was taken, synchronous and asynchronous stimulations were delivered over a 2-min-long stroking period. The pattern and frequency of the stimulation (1 Hz) were the same in both conditions due to the use of a metronome that guided the experimenter through an earphone. Directly after the stimulation (both synchronous and asynchronous stroking), the proprioceptive drift and the subjective feeling of ownership were assessed. The strength of the proprioceptive drift was defined as the distance between the two reported locations of the index finger reported in the baseline and the stimulation trials^50–52^.

#### Analysis 1 (subjective ratings)

To assess participants’ subjective perception of ownership over the rubber hand, they were asked to complete the RHI questionnaire in both the synchronous and the asynchronous conditions^6^. The original questionnaire consists of 27 items, targeting the subjective feelings occurring during the illusion, ownership, and disownership, along with some control statements. Participants had to indicate their agreement or disagreement on a 7-item Likert scale. For the current study, four questions were adopted from the RHI questionnaire. Three questions measured body ownership (*Q1. It seemed as if I were feeling the touch of the paintbrush in the location where I saw the rubber hand touched. Q2. It seemed as though the touch I felt was caused by the paintbrush touching the rubber hand. Q3 I felt as if the rubber hand was my hand*) and one further question was a control item to test the response bias (*Q4. It seemed as if I might have two right hands or arms*). The 7-item Likert scale was changed to an 11-item scale as it was applied in several RHI studies (e.g.^50,52^). Participants responded to each statement by choosing a number ranging from 0 (‘strongly disagree’) to 10 (‘strongly agree’). The reliability of the three test questions was tested for both hands in the synchronous and asynchronous conditions (right hand synchronous condition: Crombach’s alpha = 0.877; right hand asynchronous condition: Crombach’s alpha = 0.672; left hand synchronous condition: Crombach’s alpha = 0.826; left hand asynchronous condition: Crombach’s alpha = 0.799). The scores on the questions assessing body ownership (Q1–Q3) were used to calculate an illusion index by adding up the differences between the synchronous and asynchronous conditions computed separately for the right and left hands (see the formula below):

$$\left[ {\left( {Q1Rightsync - Q1Rightasync} \right) + \left( {Q2Rightsync - Q2Rightasync} \right) + \left( {Q3Rightsync - Q3Rightasync} \right)} \right] + \left[ {\left( {Q1Leftsync - Q1Leftasync} \right) + \left( {Q2Leftsync - Q2Leftasync} \right) + \left( {Q3Leftsync - Q3Leftasync} \right)} \right].$$

Results greater than zero indicated that the participant experienced a greater RHI during the synchronous condition than the asynchronous condition. The illusion index was used in the statistical analysis to investigate which of the brain structures are related to body ownership.

#### Analysis 2 (proprioceptive drift)

A similar method was used to calculate a proprioceptive drift index:

$$\left( {RightSyncDrift - RightAsyncDrift} \right) + \left( {LeftSyncDrift - LeftAsyncDrift} \right).$$

### MRI data collection and analysis

MRI data collection and the analysis of MRI measurements were performed on a 3 Tesla MR scanner (Siemens Magnetom Trio Tim System, SiemensAG, Erlangen, Germany) with a 12-channel head coil. Isotropic 3D T1-weighted sagittal magnetization-prepared rapid acquisition with gradient echo images were used for the volumetric analysis (repetition time = 2530 ms, echo time = 3.37 ms, inversion time = 1100 ms, slice thickness = 1 mm, slice number = 176, flip angle = 7°, receiver bandwidth = 200 Hz/pixel, field of view = 256 × 256 mm^2^, 256 × 256 matrix).

For the cortical reconstruction and volumetric segmentation of the images, Freesurfer v6.0 was used (http://surfer.nmr.mgh.harvard.edu/). This software allows us to assess the thickness of predefined brain structures in a large number of subjects^53,54^. Freesurfer’s semi-automatic anatomical processing scripts (autorecon 1, 2, and 3) were executed on all MR measurements. A visual check was performed for all subjects by an experienced MR expert who participated in our previous studies^50,55^. Error corrections were applied when indicated according to the recommended pipeline (https://surfer.nmr.mgh.harvard.edu/fswiki/RecommendedReconstruction) with either changing the thresholds or adding reference points to assist the software to conduct a more precise evaluation of the images. In total, four participants’ images required corrections. Since the participants were healthy and young university students, they all tolerated well the experimental situation and no motion was observed on the images. Along with this, no participants were excluded due to poor data quality. We conducted the analysis of 16 brain areas bilaterally, which were selected based on previous body ownership-related researches: STG^39,40^, MTG^41^, inferior temporal gyrus^42^, precuneus^39,40^, SMG^19,20^, precentral and postcentral gyri^18^, superior parietal gyrus^43,44^, inferior parietal gyrus^45^, lateral and medial OFC^46^, rostral anterior cingulate cortex^18^, pars opercularis^45,47,48^, LOC^14^, and insula^3^.

### Statistical analysis

Data analysis was performed using IBM SPSS Statistics for Windows, version 22.0 (IBM Corp., Armonk, NY, USA). A p value of less than 0.05 was considered statistically significant. Repeated measures ANOVAs (rANOVAs) with within-subject factors of Side (right vs. left) and Condition (synchronous vs. asynchronous) were performed for the mean questionnaire score (i.e., the mean of scores on Q1, Q2, and Q3) and the proprioceptive drift. Effect sizes were calculated using partial eta squared (η_p_^2^).

The associations between body ownership (proprioceptive drift and body ownership) and cortical thickness were analyzed by a series of multiple linear regression analysis. We created separate models for each brain structure, in which body ownership indexes were the dependent variables and the cortical thickness of the brain structures were the independent variables. In addition, age, biological sex and head size (i.e.: intracranial volume (ICV)^55^) were entered as covariates into the models.

Proprioceptive drifts related to left and right hemispheres were evaluated separately. Since some of the regions of interest showed significant association with ICV, head size correction was carried out by entering participants’ ICV as an additional independent variable into the models^55^. To ensure the assumptions of linear regression were not violated, we followed the guidelines of^56^. Normality of residuals was assessed upon QQ plot inspection. Residuals were examined for the presence of outliers (i.e. residuals outside ± 3 SDs) too. Independence of errors was tested by the Durbin-Watson test. Multicollinearity was assessed using the variance inflation factor (VIF, i.e.: VIF values under 10 indicated the absence of multicollinearity). Homoscedasticity was tested by the Breusch-Pagan test. The Benjamini–Hochberg false discovery rate (FDR) procedure was used to correct for multiple testing^57–59^; the q was set at 10%. Based on previous results our main interests were the right hemispheric brain areas, but with an exploratory purpose we analyzed the left hemispheric areas as well. Therefore, we applied the Benjamini–Hochberg correction on the analyzes of the right and the left hemispheric areas separately (Supplementary material).

## Results

### Behavioral analysis

#### Analysis 1 (subjective ratings)

When investigating the mean questionnaire scores, the main effect for the condition was significant (F(1,66) = 92.492, p < 0.001, η_p_^2^ = 0.584). Participants reported a significantly stronger illusion in the synchronous condition than in the asynchronous one. The mean questionnaire scores were higher in the synchronous condition on both the right (synchronous condition: mean = 13.112 SD = 8.28; asynchronous condition: mean = 5.88 SD = 5.65) and the left hands (synchronous condition: mean = 14.179 SD = 7.75; asynchronous condition: mean = 7.463 SD = 7.01). The main effect for Side was not significant (F(1,66) = 2.942, p = 0.091). The Condition x Side interaction was also not significant (F(1,66) = 0.258, p = 0.613).

We also analyzed if participants scored higher on the body ownership questions than on the control question using a paired sample t test. For the analysis, we used an average test question score which we calculated from the aforementioned illusion index divided by three; the scores on the control questions were integrated in a similar way:

$$\left( {Q4Rightsync \, - \, Q4Rightasynch} \right) + \left( {Q4Leftsynch \, - \, Q4Leftasynch} \right).$$

The analysis revealed that participants scored significantly higher on the test questions than on the control questions (t(66) = − 6.313 p < 0.001 Cohen’s d = 0.771; test questions mean score = 4.652 SD = 3.96; control questions mean score = 1.015 SD = 3.27).

For the analysis between proprioceptive drift and the subjective feeling of ownership, we used Spearman’s Correlation as the data did not fulfill bivariate normality. The analysis showed a positive correlation between the two variables (r = 0.305, p = 0.013).

#### Analysis 2 (proprioceptive drift)

**Figure 1:**
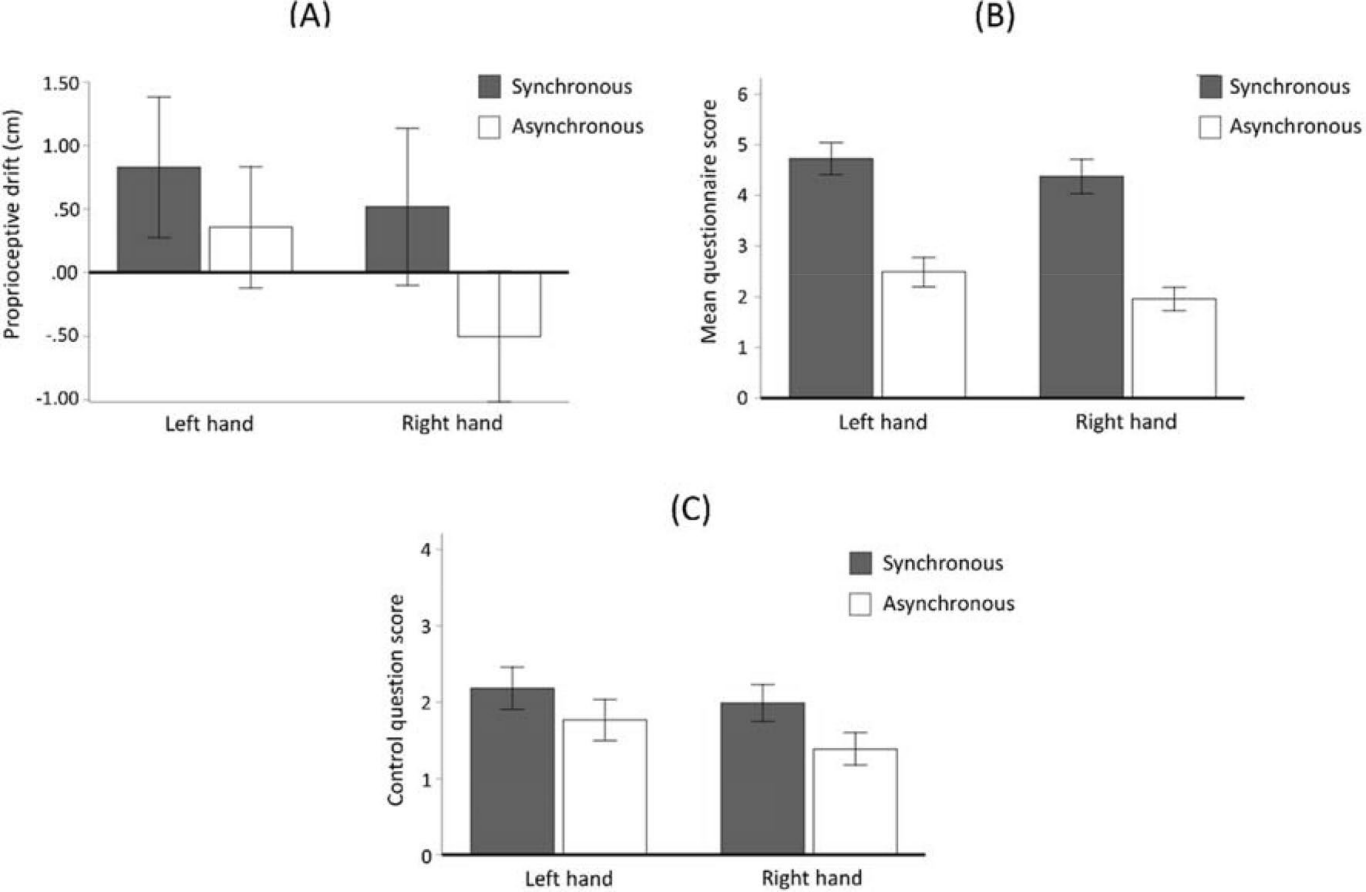
Descriptive statistics of proprioceptive drift (**A**), mean questionnaire scores (**B**) and the control question score (**C**) in the four experimental conditions. Error bars represent ± 1 standard error.

For the analysis of proprioceptive drift, we used a 2 × 2 rANOVA in which the main effect for the condition was significant (F(1,66) = 4.582, p = 0.036, η_p_^2^ = 0.066). The mean drift was higher in the synchronous condition on both the right (synchronous condition: mean = 0.518 SD = 5.02; asynchronous condition: mean = − 0.503 SD = 4.17) and the left hands (synchronous condition: mean = 0.827 SD = 4.50; asynchronous condition: mean = 0.356 SD = 3.86, see Fig. 1.). The main effect for Side was not significant (F(1,66) = 1.516, p = 0.223). The Condition x Side interaction was also not significant (F(1,66) = 0.841, p = 0.363).

### Cortical thickness and body ownership

#### Analysis 1 (subjective ratings)

**Figure 2:**
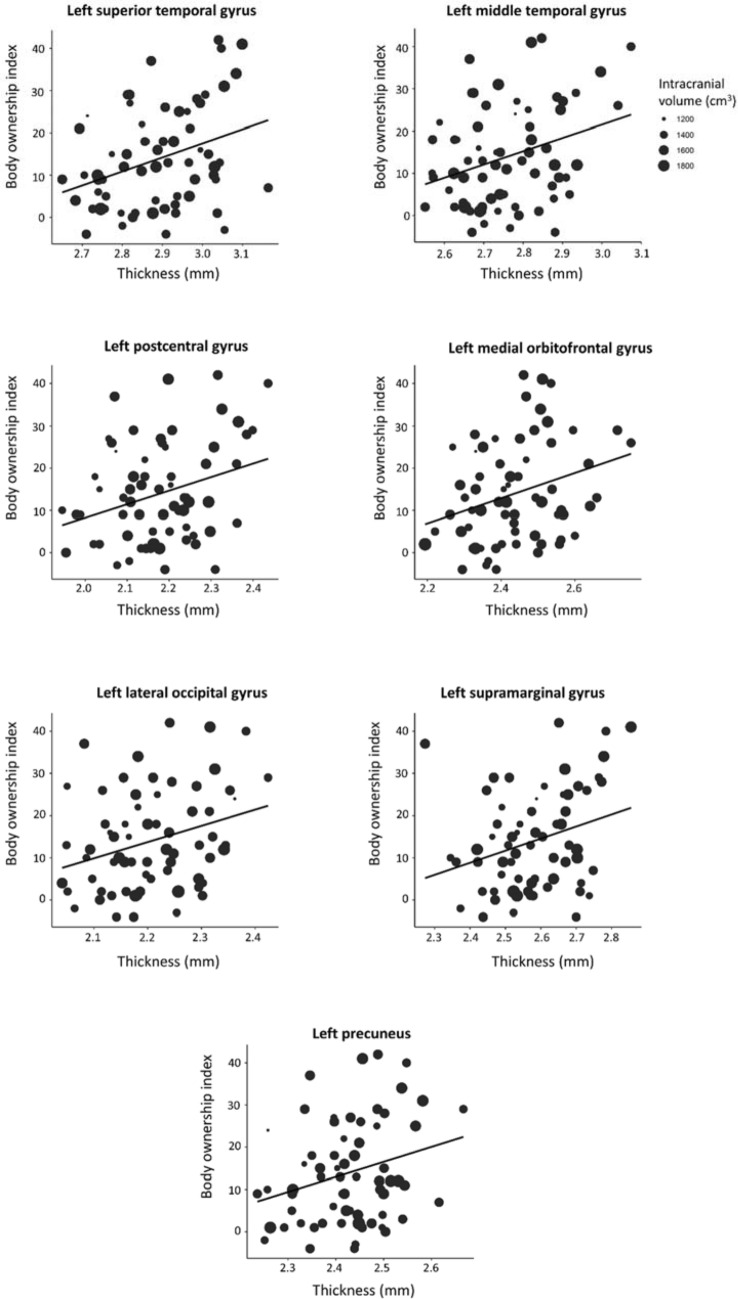
Significant associations between rubber hand illusion measures and cortical thickness of brain areas.

Body ownership index was positively associated with the cortical thickness of the bilateral MTG, the bilateral STG, the bilateral postcentral gyrus, the left LOC, the bilateral precuneus, the bilateral SMG, the left medial OFC, and the right insula (see Table 1 and Fig. 2. for summary). All the left hemispheric brain structures (MTG, STG, postcentral gyrus, LOC, precuneus, SMG, and the medial OFC) survived the Benjamini–Hochberg correction.

**Table 1:** The associations between body ownership index and cortical thickness.

Area	Hemisphere	Standardized β	t	Uncorrected p
Inferior parietal gyrus	Left	.159	1.324	.190
	Right	.162	1.289	.202
Inferior temporal gyrus	Left	.199	1.690	.096
	Right	.187	1.585	.118
Insula	Left	.296	2.514	.015*
	Right	.187	1.512	.136
Lateral occipital gyrus	Left	.144	1.137	.260
	Right	.131	1.043	.301
Lateral orbitofrontal gyrus	Left	.144	1.140	.260
	Right	.131	1.043	.301
Medial orbitofrontal gyrus	Left	.254	2.128	.037*
	Right	.102	.824	.413
Middle temporal gyrus	Left	.256	2.129	.037*
	Right	.225	1.762	.083
Pars opercularis	Left	.015	.124	.901
	Right	.178	1.465	.148
Postcentral gyrus	Left	.341	2.886	.005*
	Right	.264	2.184	.033
Precentral gyrus	Left	.198	1.580	.119
	Right	.125	1.014	.315
Precuneus	Left	.278	2.324	.023*
	Right	.252	2.080	.042
Rostral anterior cingulate	Left	.139	1.129	.263
	Right	.080	.657	.513
Superior parietal gyrus	Left	.217	1.778	.080
	Right	.228	1.938	.057
Superior temporal gyrus	Left	.380	3.410	.001*
	Right	.288	2.457	.017
Supramarginal gyrus	Left	.248	2.082	.041*
	Right	.226	1.855	.068

All estimates were controlled for age, sex and intracranial volume.

* Survived the Benjamini–Hochberg procedure (FDR was set at 10%).

#### Analysis 2 (proprioceptive drift)

Proprioceptive drift index was negatively associated with the cortical thickness of the left transverse temporal- and, the bilateral precentral gyri, and the right MTG; however, only the left transverse temporal gyrus survived the Benjamini–Hochberg correction (see Table 2 for summary).

**Table 2:** The associations between proprioceptive drift index and cortical thickness.

Area	Hemisphere	Standardized β	t	Uncorrected p
Inferior parietal gyrus	Left	− .140	− 1.135	.261
	Right	− .241	− 1.898	.063
Inferior temporal gyrus	Left	− .189	− 1.574	.121
	Right	− .162	− 1.334	.187
Insula	Left	− .191	− 1.546	.127
	Right	− .130	− 1.051	.298
Lateral occipital gyrus	Left	− .086	− .684	.497
	Right	− .151	− 1.188	.239
Lateral orbitofrontal gyrus	Left	− .119	− .914	.365
	Right	− .127	− .990	.326
Medial orbitofrontal gyrus	Left	.040	.316	.754
	Right	− .204	− 1.632	.108
Middle temporal gyrus	Left	− .155	− 1.216	.229
	Right	− .436	− 3.621	< .001*
Pars opercularis	Left	− .170	− 1.357	.180
	Right	− .036	− .281	.780
Postcentral gyrus	Left	− .012	− .093	.926
	Right	− .013	− .105	.917
Precentral gyrus	Left	− .176	− 1.344	.184
	Right	− .201	− 1.619	.111
Precuneus	Left	− .120	− .950	.346
	Right	− .227	− 1.829	.072
Rostral anterior cingulate	Left	− .054	− .423	.674
	Right	− .212	− 1.749	.085
Superior parietal gyrus	Left	− .034	− .266	.791
	Right	− .087	− .707	.482
Superior temporal gyrus	Left	− .107	− .861	.393
	Right	− .120	− .969	.336
Supramarginal gyrus	Left	− .074	− .581	.563
	Right	− .287	− 2.349	.022

All estimates were controlled for age, sex and intracranial volume.

* Survived the Benjamini–Hochberg procedure (FDR was set at 10%).

## Discussion

The aim of this study was to investigate whether the inter-individual differences of body ownership observed in RHI reflect the anatomical brain structure in terms of cortical thickness. We evoked the RHI in an experimental paradigm to measure both the subjective and objective aspects of body ownership. The behavioural analysis of the RHI, in line with previous studies^1,3,4^, illustrated that the subjective feeling of ownership was significantly stronger on both right and left hands after synchronous stimulation compared to asynchronous stimulation. With regard to the proprioceptive drift, we observed a significant difference between synchronous and asynchronous stimulations for both hands, as per our expectations. As suggested by the extant literature^4,5,7^ we also found a positive correlation between the subjective body ownership experience and the proprioceptive drift, although this was a weak association.

We found a positive relationship between the self-report of the subjective experience of body ownership during the RHI and the cortical thickness of the left LOC (considered as part of the EBA), bilateral MTG, and the bilateral precuneus. The EBA is a region selectively involved in the visual processing of the whole body^14^ and body parts^60,61^, but not faces^14^. It is suggested to be involved in the identification of individuals in situations in which the face is not visible or recognizable^14^. The EBA is also considered to be part of the left-lateralized praxis representation network (including regions of the parietal, frontal, and temporal cortex) and contributes to the semantic processing, as the EBA is sensitive to the meaning of symbolic gestures^62,63^. In more detail, the EBA with the left MTG shows activation for the visual processing of hand gestures providing the meaning of actions and representing the semantic ‘what’ of visually processed actions^41^.

Self-report scores on body ownership questions were positively correlated with the cortical thickness of the bilateral postcentral gyrus, SMG, STG, and the precuneus, which have previously been shown to be related to body ownership experience^39,40^. The subjective experience of body ownership is facilitated by the activation of the STG and SMG; these areas are known to be involved in the sense of agency as well, that is strongly related to body ownership^64^. The precuneus is also involved in self-other differentiation and is a part of a self-referential network, along with the superior frontal gyrus and the posterior cingulate^46^.

The subjective feeling of ownership positively correlated with the cortical thickness of the right insula, which has a pivotal role in multisensory integration^21^. This finding is in line with previous studies, which have indicated a positive correlation between the right insula and the strength of the RHI assessed by self-report (e.g.^3^). With the exception of its involvement in hand ownership, the right insula is part of a right-hemispheric predominant brain network which encodes two components of self-consciousness: self-location and first-person perspective^65^. In addition, we found the existence of a positive relationship between the self-reports of body ownership and the cortical thickness of the left medial OFC. The orbitofrontal region is part of an anatomical and functional unit, called the cortical midline structures (CMS)^46^. The CMS are involved in self-referential processing, which is suggested to play an intermediary role between sensory and higher-order cognitive processes^66^.

Our findings show that the individual differences of the two body ownership measurements induced by RHI are associated with the structural anatomy of different brain areas. More specifically, proprioceptive drift was negatively correlated with the cortical thickness of regions such as the precentral gyrus, the transverse temporal gyrus, and the MTG, although only the transverse temporal gyrus survived the Benjamini–Hochberg correction. The aforementioned areas have been associated with multisensory integration^18^, auditory processing, sensory processing, and higher-order self-related functions^18,41^. On the other hand, the subjective experience of body ownership intensity was correlated with the cortical thickness of areas that are linked to multisensory integration processing (STG, SMT)^10,19^, the visual processing of the body (LOC)^14^, and higher order self-related functions (OFC and MTG)^41,46^. The analysis of cortical thickness supports the dissociation of the subjective and objective aspects of the RHI.

Previous studies showed, that the precentral gyrus is an important contributor to the body ownership experience over the rubber hand^67^. In line with this the majority of fMRI studies focusing on bodily illusions emphasized the link between the precentral gyrus (mostly the premotor cluster of it) and the subjectively rated strength of ownership of a fake/virtual body or body parts^1,13,68^, thus suggesting that the activity in this area reflects changes in the subjective feeling of body ownership. Interestingly, however, we failed to identify a relationship between the thickness of the left precentral region and the strength of the illusion, as measured by the questionnaire. Instead, the thickness of the bilateral precentral area was negatively associated with the proprioceptive drift. The precentral gyrus is known to be part of a region where the number of “peripersonal neurons” is high, and it is usually referred to as the *polysensory zone*^69^. The neurons in this area respond to visual, tactile, and auditory stimuli, and also play a role in defensive behavior within the peripersonal space^70,71^.

Additionally, we found a negative correlation between the thickness of the right MTG and the proprioceptive drift. The MTG is known to have a role in the visual processing of hand gestures^41^. According to our results, it might be the case that those who have a smaller cortical thickness in the MTG have difficulties with the visual processing of hand movements, which would result in a larger proprioceptive drift. In the absence of more convincing evidence, however, this explanation should be treated with caution.

Besides its significant contributions, the current study has a few limitations. With respect to the subjective feeling of body ownership and the proprioceptive drift, we found significant correlations with the cortical thickness of multiple brain areas; however, very few of them survived the Benjamini–Hochberg correction. Moreover, this study was correlational in nature and provides no insight into the causal relationship between the measured factors. Furthermore, our participants were all young university students, hence these results cannot be generalized to the entire population. In addition, due to the high variability on our data it would be beneficial to expand our research and include more participants in the study. On this note, the results need to be interpreted with caution. Nonetheless, this study may still shed light on new perspectives for further considerations in body ownership research (e.g., studying clinical populations).

## Conclusion

In sum, our findings support the view that the inter-individual differences of the body ownership experience may not just appear in differences in brain function but are also pronounced in the anatomical brain structure in terms of cortical thickness. In the present study, we investigated the objective and the subjective aspects of body ownership, both of which were found to be associated with the cortical thickness of areas previously shown to be functionally related to body ownership. The structural correlates of proprioceptive drift (i.e., the objective aspect) involved areas previously reported to be responsible for multisensory integration, action and movement perception, and body awareness; on the other hand, the subjective experience of body ownership correlated with the structural measures of areas involved in visual processing of the body, multisensory integration processing, somatosensory processing, and higher-order functions. Thus, our results are in line with the findings of functional neuroimaging studies and suggest that individual differences in body ownership experiences can also manifest in differences in the anatomic brain structure.

## Supplementary Information

Supplementary Information.
