# Supplementary material for: Individual differences in the experience of body ownership are related to cortical thickness
Source: Sci Rep. 2022 Jan 17;12:808. doi: 10.1038/s41598-021-04720-8 (PMC8764083; doi:10.1038/s41598-021-04720-8)
Supplement: Supplementary file 1 — Supplementary Information. [file 41598_2021_4720_MOESM1_ESM.pdf]

Individual differences in the experience of body ownership are related to cortical thickness

Timea Matuz-Budai, Beatrix Lábadi, Eszter Kohn, András Matuz, András Norbert Zsidó, Orsolya Inhóf, János Kállai, Tibor Szolcsányi, Gábor Perlaki, Gergely Orsi, Szilvia Anett Nagy, József Janszky, Gergely Darnai

Supplementary material

Statistical analysis

Correction for multiple comparisons

To adjust for multiple testing across the correlation matrices, we applied the Benjamini-Hochberg procedure with an FDR rate of 10%. This procedure involves ranking the p-values from the significance tests from the smallest to the largest. The formula *(i/m)*FDR* yields a Benjamini-Hochberg critical value for each test.

The rank number is „i"(it ranges from 1 to 15).

The number of significance tests is „m" (in our case 15)

The chosen FDR is „FDR" (in our case 0.1).

Results were considered significant when p-values were smaller than the threshold obtained from the equation.

In our manuscript we report both the p-values that remained significant after correction and those ones which were not retained after the correction.

The Benjamini-Hochberg corrected p-values were calculated using the spreadsheet available from here:

http://www.biostathandbook.com/benjaminihochberg.xls

The description about the correction method available here:

http://www.biostathandbook.com/multiplecomparisons.html
